# Dissection of the bone marrow microenvironment in hairy cell leukaemia identifies prognostic tumour and immune related biomarkers

**DOI:** 10.1038/s41598-021-98536-1

**Published:** 2021-09-24

**Authors:** Rachel M. Koldej, Ashvind Prabahran, Chin Wee Tan, Ashley P. Ng, Melissa J. Davis, David S. Ritchie

**Affiliations:** 1 ACRF Translational Research Laboratory, Royal Melbourne Hospital, Melbourne, Australia; 2 Department of Medicine, Faculty of Medicine, Dentistry and Health Sciences, University of Melbourne, Melbourne, Australia; 3 Clinical Haematology, Peter MacCallum Cancer Centre and Royal Melbourne Hospital, Melbourne, Australia; 4 The Walter and Eliza Hall Institute of Medical Research, Parkville, Melbourne, Australia; 5 Department of Medical Biology, Faculty of Medicine, Dentistry and Health Sciences, University of Melbourne, Melbourne, Australia; 6 Department of Clinical Pathology, Faculty of Medicine, Dentistry and Health Sciences, University of Melbourne, Melbourne, Australia

**Keywords:** Bioinformatics, Immunohistochemistry, Prognostic markers, Translational research, Cancer microenvironment, Haematological cancer, Tumour immunology

## Abstract

Hairy cell leukaemia (HCL) is a rare CD20+ B cell malignancy characterised by rare “hairy” B cells and extensive bone marrow (BM) infiltration. Frontline treatment with the purine analogue cladribine (CDA) results in a highly variable response duration. We hypothesised that analysis of the BM tumour microenvironment would identify prognostic biomarkers of response to CDA. HCL BM immunology pre and post CDA treatment and healthy controls were analysed using Digital Spatial Profiling to assess the expression of 57 proteins using an immunology panel. A bioinformatics pipeline was developed to accommodate the more complex experimental design of a spatially resolved study. Treatment with CDA was associated with the reduction in expression of HCL tumour markers (CD20, CD11c) and increased expression of myeloid markers (CD14, CD68, CD66b, ARG1). Expression of HLA-DR, STING, CTLA4, VISTA, OX40L were dysregulated pre- and post-CDA. Duration of response to treatment was associated with greater reduction in tumour burden and infiltration by CD8 T cells into the BM post-CDA. This is the first study to provide a high multiplex analysis of HCL BM microenvironment demonstrating significant immune dysregulation and identify biomarkers of response to CDA. With validation in future studies, prospective application of these biomarkers could allow early identification and increased monitoring in patients at increased relapse risk post CDA.

## Introduction

Hairy cell leukaemia (HCL) is an uncommon CD20+ B cell malignancy, accounting for < 2% of leukemias, and is characterised by rare circulating B cells with cytoplasmic, villous projections, splenomegaly and extensive bone marrow (BM) infiltration with resultant pancytopenia. Standard front line therapy with the purine analogue cladribine (CDA) either alone or in combination with an anti-CD20 antibody immunotherapy has an initial response rate of > 70%^1,2^. However, the duration of response is highly variable with many patients relapsing within 10 years.

While studies of HCL biology have largely focused on analysis of surface marker expression on pathogenic HCL cells^3–5^ including expression of immunomodulators such as PD-1^6^, studies investigating the immune environment associated with HCL are limited. Early studies did demonstrate deficiencies in T and NK cell activity^7,8^ with T cells from HCL patients showing impaired proliferative responses, likely due to variation in the expression of CD28, and subsequent restricted T cell repertoire^9^. Alterations in CD4 T cell memory subsets have been described both at diagnosis^10^ and post-CDA^11^. There has been a paucity of studies in HCL immunology in recent years. Whether immune responses, either at diagnosis or post-CDA, contribute to long term disease control is unknown. Similarly, greater understanding of the extent of post-CDA residual immunity may also allow the informed application of immunotherapies to effect more durable remissions in HCL.

Analysis of the immunobiology of HCL is complicated by its low incidence, the low number of circulating tumour cells and that sampling of the site of the bulk of the tumour requires sampling of the BM. Furthermore, at diagnosis many patients have marrow fibrosis resulting in very limited, if any, material available from BM aspirates^12,13^. While routine archival samples of BM trephines are collected from all patients, due to acid decalcification during processing, they are not amenable to immune profiling by gene expression signature and can only be used for protein analysis. We have recently shown that NanoString GeoMX™ Digital Spatial Profiling (DSP) can be used to analyse high multiplex protein expression in BM trephine samples^14^. In this current study, we have utilised DSP to examine the immune microenvironment in HCL pre- and post-CDA to provide a detailed analysis of the marrow microenvironment and determine biomarkers of durable response to CDA which could therefore be applied prospectively to identify patients that are at risk of relapse post-CDA and require further monitoring.

## Results

### Pre- and post-CDA changes in HCL tumour microenvironment

**Figure 1:**
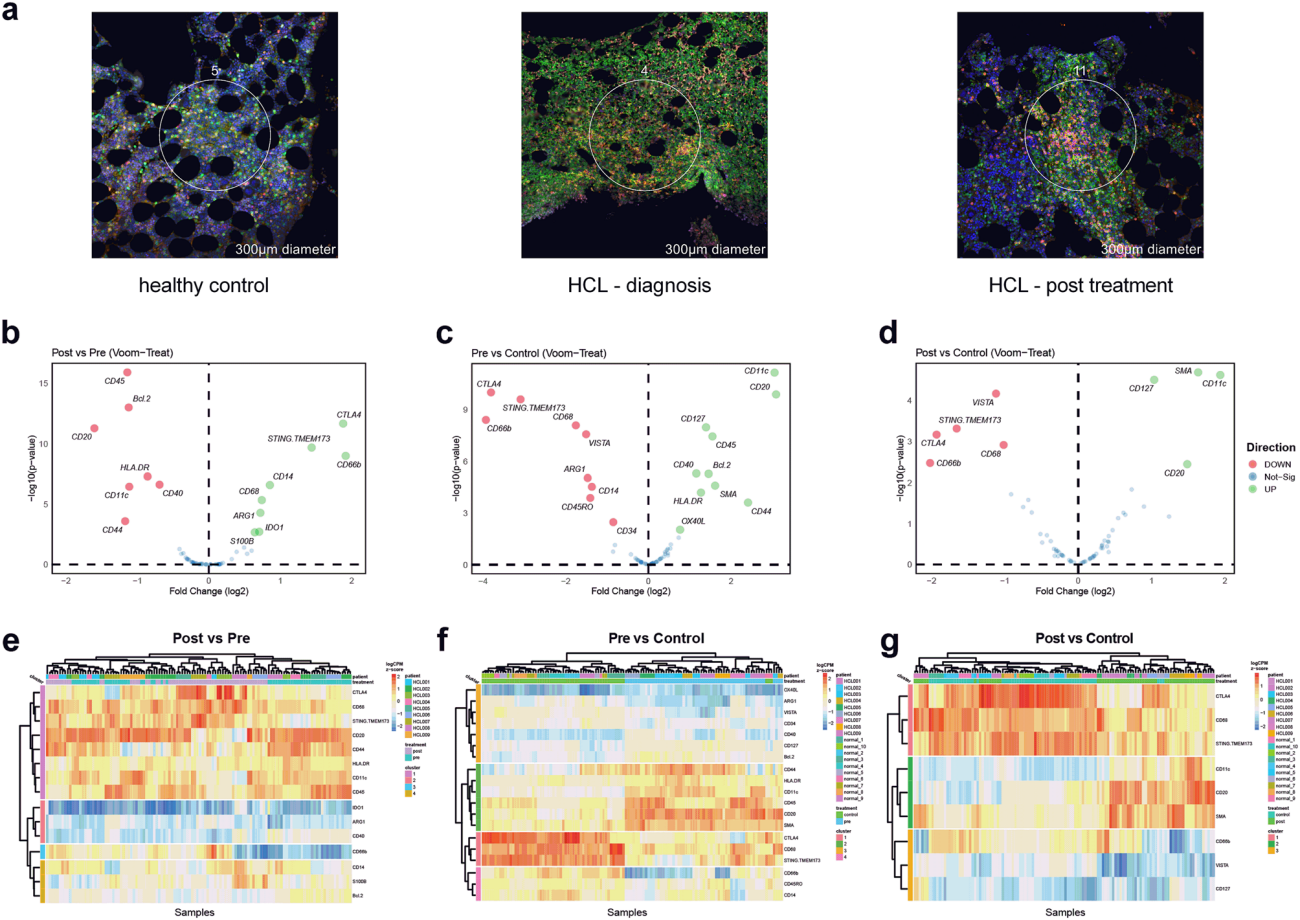
DSP identifies changes in multiple immune markers between pre-CDA, post-CDA and control samples. (**a**) Representative ROIs from each sample type in this study. Red = CD3, Green = CD45, Blue = nuclei, Yellow = dual CD3/CD45. (**b**)–(**d**) Results of limma-voom-treat multivariate analysis comparing differential expression between pre- versus post-CDA (**b**), pre-CDA versus control (**c**) and post-CDA versus control (**d**). Heatmap of respective samples and differential expression markers for the following comparison: pre- versus post-CDA (**e**), pre-CDA versus control (**f**) and post-CDA versus control (**g**). Z-score normalised logCPM visualised in each heatmap. Heatmaps were generated in R (version 1.0.12) using R package pheatmap. Hierarchical clustering was conducted using the hclust function in the base R statistics package.

Analysis of 9 HCL patient BM trephine samples pre and post-CDA using a pre-designed DSP panel (Fig. 1a) identified that fifteen markers were differentially expressed (8 up- and 7 down-regulated) when corrected for multiple comparisons and normalised to the total nucleated cells within a field of view. The most significant downregulated proteins identified were CD45, BCL-2 and CD20: likely reflecting changes in HCL tumour burden (Fig. 1b,e and Table 1). Compared to 10 healthy control samples, pre- and post-CDA patients exhibited significant differential expression of 19 (10 up- and 9 down-regulated) and 9 (4 up- and 5 down-regulated) surface markers respectively (Fig. 1c,d,f,g and Table 1). Overall this suggests that there are greater differences in the tumour and immune landscape between pre-CDA samples and post-CDA/healthy control samples. While post-CDA samples show normalisation of multiple markers to healthy control levels, there is ongoing differential expression of multiple markers.

**Table 1:** Multivariate analysis results.

Analysis	Marker	logFC	AveExpr	*p* value	adj. *p* val
**Post versus Pre**	CD45	− 1.142	14.43	1.22E−16	7.81E−15
	Bcl.2	− 1.122	11.8	9.95E−14	3.18E−12
	CTLA4	1.878	15.05	2.19E−12	4.66E−11
	CD20	− 1.602	14.88	5.30E−12	8.48E−11
	STING.TMEM173	1.438	15.09	2.12E−10	2.72E−09
	CD66b	1.914	12.49	1.04E−09	1.11E−08
	HLA.DR	− 0.8581	13.53	5.13E−08	4.69E−07
	CD40	− 0.6905	10.81	2.39E−07	1.83E−06
	CD14	0.8537	13.28	2.58E−07	1.83E−06
	CD11c	− 1.114	13.59	3.54E−07	2.26E−06
	CD68	0.74	15.48	4.59E−06	2.67E−05
	ARG1	0.7202	11.01	5.2E−05	0.000277
	CD44	− 1.172	13.71	0.0003	0.0013
	IDO1	0.7039	8.896	0.0019	0.0089
	S100B	0.6422	12.52	0.0022	0.0092
**Pre versus Healthy Control**	CD11c	3.051	13.59	6.94E−12	4.44E−10
	CTLA4	− 3.809	15.05	9.86E−11	2.75E−09
	CD20	3.088	14.88	1.29E−10	2.75E−09
	STING.TMEM173	− 3.094	15.09	2.49E−10	3.98E−09
	CD66b	− 3.933	12.49	3.93E−09	5.02E−08
	CD68	− 1.756	15.48	8.13E−09	8.67E−08
	CD127	1.393	12.12	1.05E−08	9.63E−08
	VISTA	− 1.508	11.68	2.68E−08	2.14E−07
	CD45	1.549	14.43	3.58E−08	2.55E−07
	CD40	1.159	10.81	4.97E−06	3.05E−05
	Bcl.2	1.455	11.8	5.23E−06	3.05E−05
	ARG1	− 1.471	11.01	9.11E−06	4.86E−05
	SMA	1.612	15.03	2.52E−05	0.0001
	CD14	− 1.369	13.28	2.94E−05	0.0001
	HLA.DR	1.27	13.53	6.35E−05	0.0003
	CD45RO	− 1.408	12.9	0.0001	0.0005
	CD44	2.409	13.71	0.0002	0.0009
	CD34	− 0.8514	11.75	0.0033	0.0119
	OX40L	0.7654	9.261	0.0091	0.0308
**Post versus Healthy Control**	SMA	1.634	15.03	2.07E−05	0.0007
	CD11c	1.936	13.59	2.4E−05	0.0007
	CD127	1.035	12.12	3.15E−05	0.0007
	VISTA	− 1.121	11.68	6.83E−05	0.0011
	STING.TMEM173	− 1.657	15.09	0.0005	0.0062
	CTLA4	− 1.931	15.05	0.0007	0.0073
	CD68	− 1.016	15.48	0.0012	0.0112
	CD66b	− 2.019	12.49	0.0034	0.0257
	CD20	1.486	14.88	0.0036	0.0257
**(Pre vs. Post for Durable) versus (Pre vs. Post for Non-Durable)**	CD20	2.181	14.88	4.95E−10	3.17E−8
	CD8	− 1.096	12.58	0.000066	0.002137
	CD3	− 1.299	13.18	0.000121	0.002582
	CD44	2.053	13.71	0.000351	0.005613
	B7H3	0.7318	12.08	0.002325	0.02976
	CTLA4	− 1.527	15.05	0.003980	0.04245

### CDA treatment effectively reduces tumour associated markers

**Figure 2:**
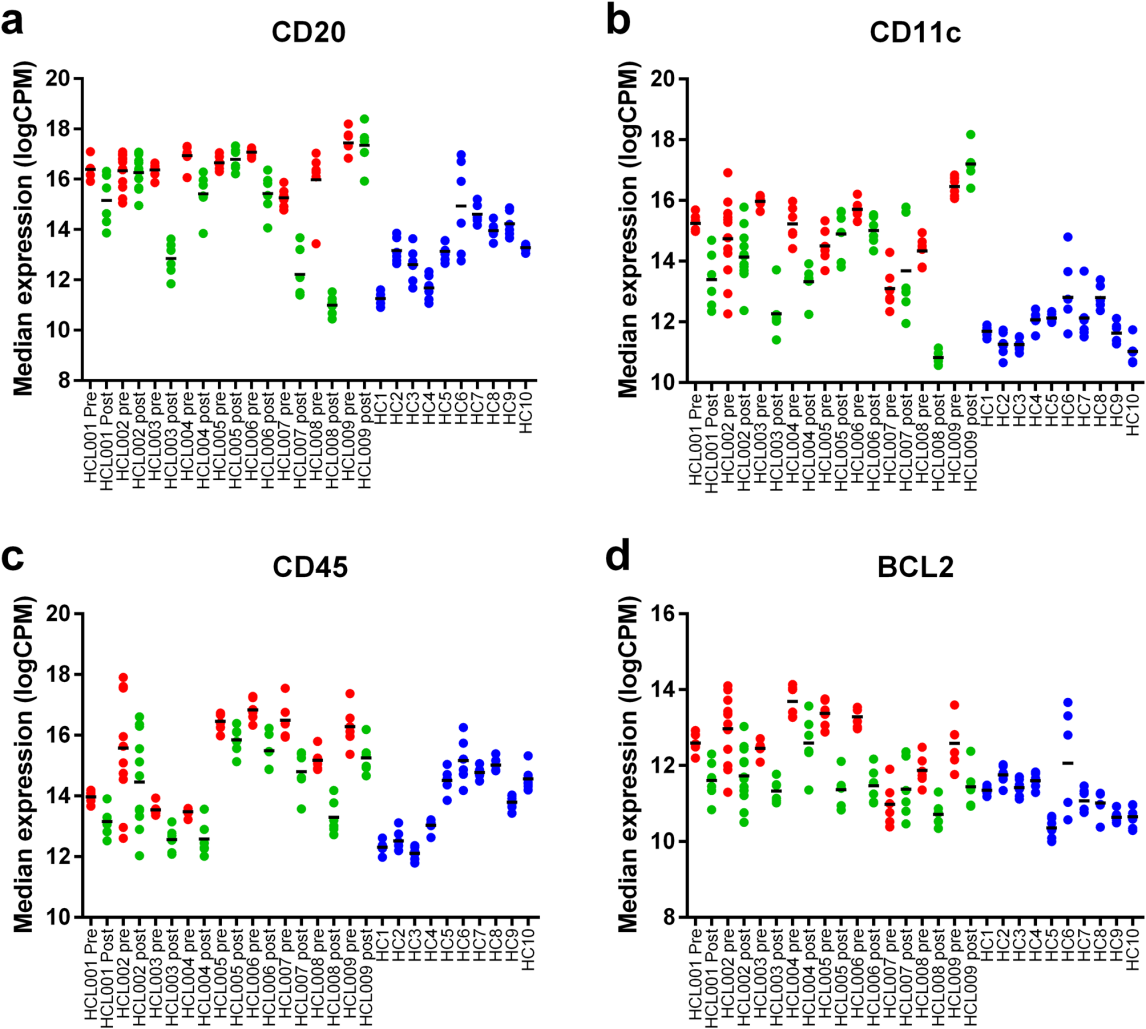
CDA treatment is associated with reduction in expression of tumour associated markers. Expression of tumour associated markers pre- (red) and post-CDA (green) compared to control samples (blue). (**a**) CD20, (**b**) CD11c, (**c**) CD45 and (**d**) BCL2.

HCL cells express multiple markers including CD20, CD11c, CD44 and CD45. Changes in CD20 and CD11c were found to be statistically significant between pre-CDA, post-CDA and healthy controls with high levels in each patient (CD20; pre vs. healthy adj *p* = 2.75 × 10^–9^, post vs. healthy adj *p* = 0.0257, pre vs. post adj *p* = 8.48 × 10^–11^. CD11c; pre vs. healthy adj *p* = 4.44 × 10^–10^, post vs. healthy adj *p* = 0.0007, pre vs. post adj *p* = 2.26 × 10^–6^). While these markers were reduced following treatment with CDA, they do not reach healthy control levels (Fig. 2a,b) suggesting the presence of residual HCL. Interestingly, for 3 patients CDA treatment did not change relative expression of CD20 or CD11c in the BM despite reductions in percentage of CD19 + cells in the PB (Supplementary Table 1). CD45 was the top differentially expressed marker pre-/post-CDA (adj *p* = 7.18 × 10^–15^), with CDA exposure reducing CD45 expression (Fig. 2c) suggesting clearance of CD45 + HCL cells, consistent with known upregulation of CD45 expression on HCL cells^5^. Interestingly, while BCL-2 was also highly expressed pre-CDA the level of this protein returned to healthy control ranges post-CDA (Fig. 2d) (pre vs. healthy adj *p* = 3.05 × 10^–5^, pre vs. post adj *p* = 3.18 × 10^–12^), likely reflecting the change in tumour burden in these patients.

### Post-CDA samples exhibit increased expression of myeloid lineage markers

**Figure 3:**
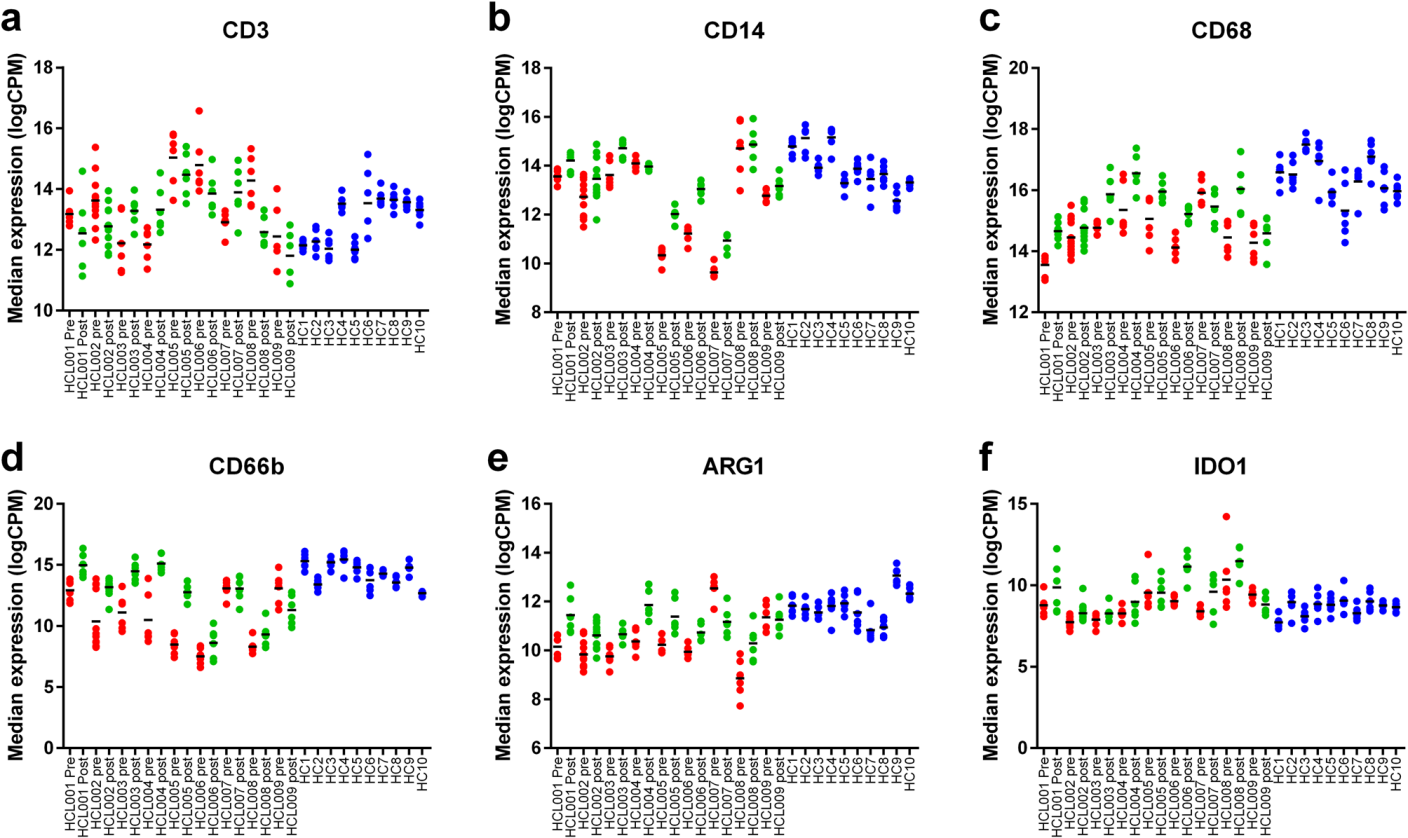
CDA treatment is associated with an increase in cells of monocytic lineages. Expression of immune cell markers pre- (red) and post-CDA (green) compared to control samples (blue). (**a**) CD3, (**b**) CD14, (**c**) CD68, (**d**) CD66b, (**e**) ARG1 and (**f**) IDO1.

The DSP panel included multiple markers to allow dissection of the immune microenvironment in the BM samples analysed. Interestingly, the relative expression of CD3, CD4, CD8 or CD56 was not significantly different pre-/post-CDA and was in the range seen in healthy controls (Fig. 3a and Supplementary Figure 1). Given the numerical decrease in BM lymphocyte count post CDA (Supplementary Table 1), this suggests that while the relative expression of T and NK makers were unchanged, there was a net loss of total T and NK cells. Correspondingly, markers associated with myeloid lineages were increased post-CDA, with increased levels of CD14 (Pre vs. healthy adj *p* = 0.0001, pre vs. post adj *p* = 1.83 × 10^–6^) and CD68 (Pre vs. healthy adj *p* = 8.67 × 10^–8^, post vs. healthy adj *p* = 0.0112, pre vs. post adj *p* = 2.67 × 10^–5^) suggesting increased proportions of monocytes and dendritic cells following therapy (Fig. 3b,c) as well as increased CD66b (Pre vs. healthy *p* = 5.02 × 10^–8^, post vs. healthy *p* = 0.0257, pre vs. post *p* = 1.11 × 10^–8^) and ARG1 (Pre vs. healthy *p* = 4.86 × 10^–5^, pre vs. post *p* = 0.0002) suggesting changes in neutrophil populations (Fig. 3d,e), and increased IDO1 (pre vs. post *p* = 0.0089) suggesting changes in dendritic cells or mesenchymal stem cells (Fig. 3f). Taken together, the relative increase in myeloid populations is likely to reflect post-CDA recovery in the setting of debulking of HCL within the BM following CDA exposure, and the relative sensitivity of lymphoid cells compared to myeloid cells to CDA treatment.

### HCL is associated with the dysregulation of MHC class-II and multiple immune checkpoints

**Figure 4:**
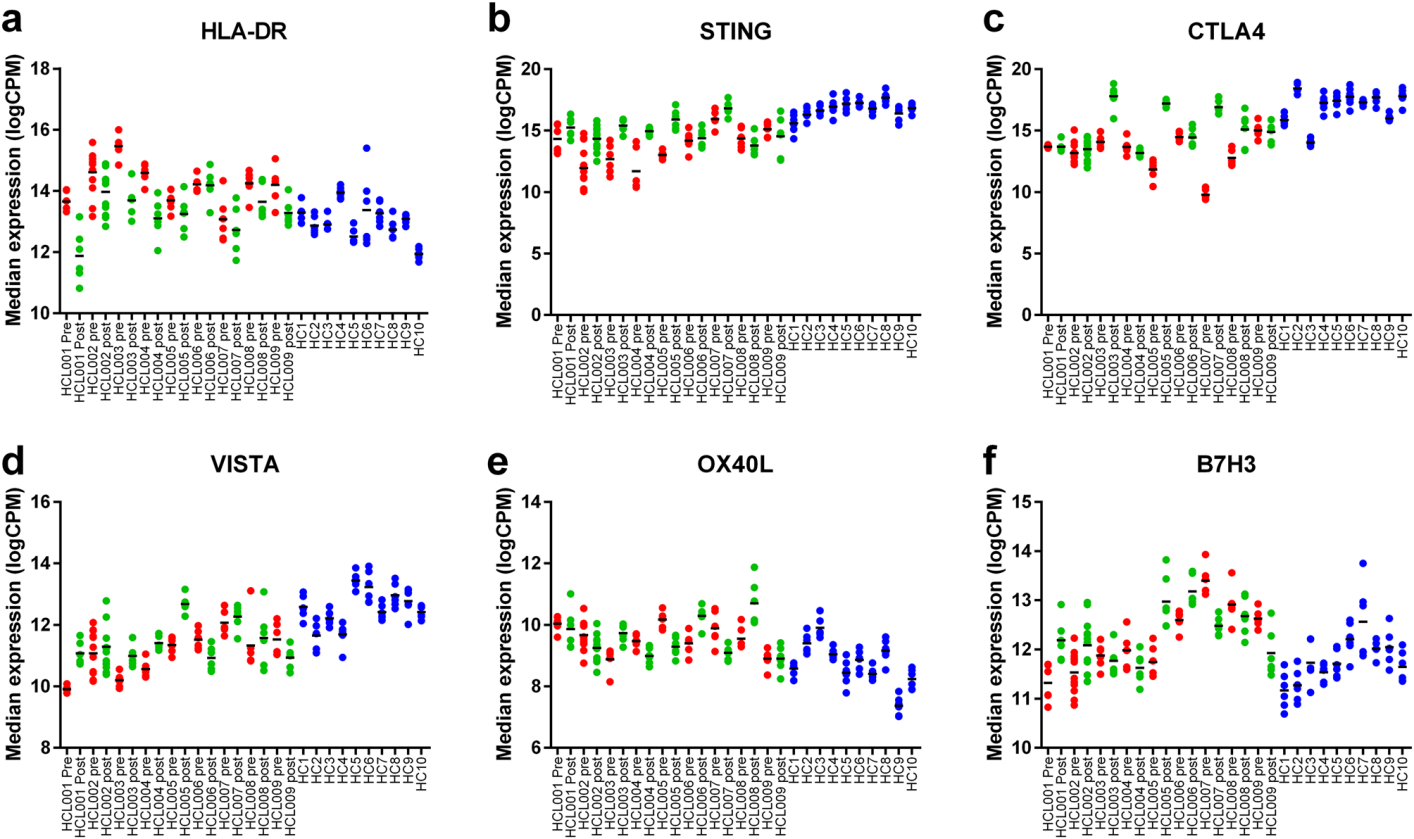
HCL patients’ exhibit altered expression in multiple immune markers that do not improve with CDA treatment. Expression of immune function markers pre- (red) and post-CDA (green) compared to control samples (blue). (**a**) HLA-DR, (**b**) STING, (**c**) CTLA4, (**d**) VISTA, (**e**) OX40L and (**f**) B7H3.

To examine the immune microenvironment in HCL, the expression of MHC class-II (HLA-DR) and multiple immune checkpoints were examined. HLA-DR was highly expressed pre-CDA and while its expression was reduced post-CDA (adj *p* = 4.69 × 10^–7^), it continued to be above that seen in healthy control samples (Fig. 4a), likely reflecting its expression in both HCL cells and monocyte lineages. The immune checkpoints STING, CTLA4 and VISTA exhibited reduced expression pre-CDA which, while increased post-CDA, was not restored to healthy control levels (STING; Pre vs. healthy adj *p* = 3.89 × 10^–9^, post vs. heathy adj *p* = 0.0062, pre vs. post adj *p* = 2.72 × 10^–9^. CTLA4; Pre vs. healthy adj *p* = 2.75 × 10^–9^, post vs. heathy adj *p* = 0.0073, pre vs. post adj *p* = 4.66 × 10^–11^. VISTA; Pre vs. healthy adj *p* = 2.14 × 10^–7^, post vs. heathy adj *p* = 0.0011). (Fig. 4b–d). In contrast, OX40L exhibited increased expression pre-CDA (adj *p* = 0.0308) which was not altered by therapy (Fig. 4e). Multiple other immune checkpoints, including B7H3 (Fig. 4f), LAG3, TIM3, PD1 and PDL1, did not show altered expression (Fig. 1). Overall, this suggests there is underlying dysregulation of multiple immune checkpoints in the immune microenvironment of HCL which is not altered by a reduction in tumour bulk following CDA therapy.

### Changes in tumour burden and T cell markers are associated with durable response to therapy

**Figure 5:**
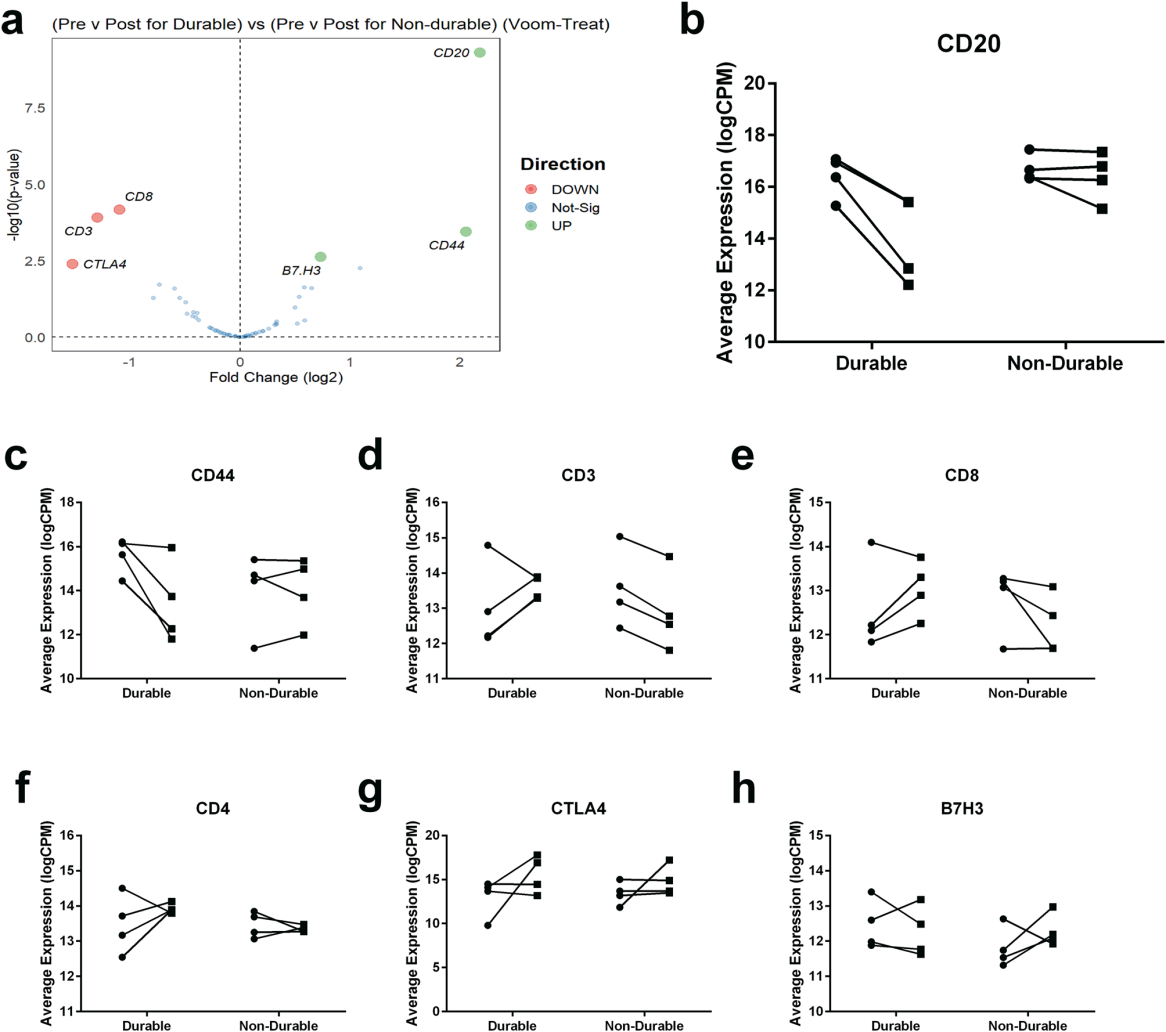
Changes in tumour burden and CD8 expression are associated with durable response to CDA. (**a**) Results of voom-limma-treat multiple comparisons of (pre vs. post for durable) versus (pre vs. post for non-durable). (**b**)–(**h**) Average marker expression pre- versus post-CDA in durable and non-durable responders. (**b**) CD20, (**c**) CD44, (**d**) CD3, (**e**) CD8, (**f**) CD4, (**g**) CTLA4, (**h**) B7H3.

To determine which protein(s) were associated with patient response to CDA, a multivariate analysis was used to examine correlation between durability of response and the change in marker expression between pre- and post-CDA samples (Fig. 5a). As would be expected, reduced expression of the B cell marker CD20 post-treatment correlated with subsequent durable clinical response (adj *p* = 3.17 × 10^–8^) as a measure of overall tumour response to CDA therapy (Fig. 5b). Reduction in CD44, which is expressed on HCL cells^15^, was also associated with durable response (adj *p* = 0.005613) (Fig. 5c) though there was no correlation with other tumour markers. This is likely due to expression of these markers on non-tumour cells (for example reduction in CD11c as a result of the reduction in tumour burden was likely offset by the increased proportions myeloid lineage cells that express CD11c).

In addition, durable responders exhibited increased expression of CD3 (adj *p* = 0.002582) and CD8 (adj *p* = 0.002137) post-CDA treatment but not CD4 (Fig. 5d–f). This relative increase in BM expression of CD3 and CD8 did not correspond with increased percentage of CD3 + cells in the PB of patients (Supplementary Table 1) suggesting increased immune infiltration into the BM by cytotoxic T cells in durable responders. Increased expression of CTLA-4 (adj *p* = 0.04245) and decreased expression of B7H3 (adj *p* = 0.02976) were also associated with CDA response (Fig. 5g–h). Taken together, this data shows that durability of response to CDA is associated with not only the degree of tumour debulking post-CDA, but also by a concurrent immune recovery in the BM, potentially resulting in additional disease control.

## Discussion

Studies into the biology of HCL have largely focused on the characterisation of the tumour cells rather than the immune microenvironment. Identification of prospective biomarkers to determine patient response have been limited by the availability of samples from HCL patients for analysis. In this study, our analysis of BM trephine samples using DSP demonstrates that there is a large, previously under-utilised sample set that can be used to study the immune microenvironment in HCL.

Digital Spatial Profiling provides much deeper picture and insights into the heterogeneity of the tumour and immune microenvironment. Applying protein analysis panels directly on the tissue sections and using standard fluorescent IHC to select regions for in depth analysis allows the analysis to be confined to areas with particular features (in this instance clusters of immune active cells) rather than the whole tissue and allows intra-tissue heterogeneity to be determined. It should be noted that as DSP uses oligonucleotide conjugated antibodies to analyse expression in each user defined region of interest, only expression in each region can be reported. It therefore does not provide cell specific expression as can be determined using other available techniques^16,17^ but does provide a higher potential multiplex (up to 96 proteins) allowing a more in-depth initial screen for biomarker discovery. Analysis of samples using DSP presents some unique statistical challenges that need to be considered. Sampling of multiple regions within a tissue, multiple samples per patient with different responses to treatment have the potential to bias the analysis if not handled appropriately. In this study, we applied a multivariate statistical approach to control for the numerous potential confounders and bias present in the protein expression dataset sampled in such a complex experimental design.

The immune sculpting effect of CDA in HCL has not been studied in detail beyond the well-known depletion of B, T and NK cells^18–20^. However, in recent years CDA has been applied to the treatment of relapsing–remitting multiple sclerosis (1.75 mg/kg per treatment course). Similar to the results of this study, immune profiling of PB subsets 3 months post-CDA has shown that the relative proportions of B cells decrease, T and NK cells remain stable and monocyte/neutrophil lineages increase^21–23^. However, when corrected for the change in lymphocyte count, there is an overall decrease in B, T and NK lineages and an increase in monocyte lineages^21^. Variation in the expression of CD28 on CD8 T cells has previously been noted in HCL patients^9^ which may impact on the ability of T cells to reconstitute post-CDA and contribute to durability of CDA response.

The relative insensitivity of dendritic cells to CDA has been demonstrated with concentrations of 2.5–7.5 nM CDA able to decrease dendritic cell proliferation without impacting on survival. CDA treated dendritic cells showed increased expression of CD86 and HLA-DR and reduced production of TNFα and IL-1β^24^. Given that decreased antigen presentation by monocytes in HCL has been hypothesised^25^, the results of the current study showing sustained increase in HLA-DR expression post-CDA despite depletion of HLA-DR expressing HCL cells suggests a net increase in antigen presenting dendritic cells/monocytes and improved immunomodulation capacity post-CDA in HCL.

Despite the change in immune subsets and potential increase in antigen presentation capacity, CDA treatment did not correct the dysregulated expression of immune checkpoints including STING, VISTA, CTLA4 or OX40L. STING regulates the production of type 1 interferons^26^ and decreased expression of STING in HCL patients may lead to decreased interferon production in the BM and explain why interferon-α has historically been shown to have some therapeutic activity in HCL^27^. Changes in the relative expression of STING and VISTA may be due to their expression on expanded monocyte populations post-CDA, whereas CTLA4 and OX40L are not expressed on monocyte populations and would not be expected to change based solely on changes in cell proportions. Indeed, changes in the expression of CTLA4, which is expressed on CD4 memory and Treg populations, correlated with patient response to treatment. Conversely, the lack of change in the expression of lymphoid specific immune checkpoints such as B7H3, LAG3 and PD1 is consistent with the total CD3 expression. Given the reduced proportions of T cells when corrected for the change in lymphocyte count, this suggests that the total number of lymphoid cells expressing these markers is reduced post-CDA. Further studies are required to dissect the relative impact of these checkpoints in the immune microenvironment of HCL.

While this analysis is limited by the patient numbers, the matched samples with multiple regions analysed per sample with long term follow up data allowed a rigorous bioinformatics analysis to be performed. This study is the first to identify potential disease prognosis biomarkers, with changes in both the proportion of CD8 T cells and degree of tumour burden clearance correlating with the duration of response to therapy in patients with HCL. Notably, this response assessment was performed on post-treatment samples taken an average of 134 days post-CDA, well before durability of clinical response was determined. Patients who go on to have short post-CDA remissions clearly have muted CD8 T cell responses, which may require novel CD8-directed therapies prior to HCL relapse to enable durable clinical responses.

With the development of DSP and the downstream bioinformatics pipelines, BM trephine samples offer a ready source of material for translational haematology research, particularly in rare diseases where correlative samples can be difficult to collect in large numbers. We have demonstrated that HCL response to CDA is dependent on both depth of tumour depletion and the immune microenvironment. Given the low sample number in this pilot study, future studies should focus on validation of these biomarkers and further dissection of the immune microenvironment in HCL to develop new diagnostic tools and therapeutic interventions in those patients likely to relapse post-CDA.

## Methods

### Patient cohort

Through a review of our centre records, 9 HCL patients treated between 2000 and 2014 with paired pre- and post-CDA treatment BM trephines were identified (mean age at diagnosis 50.2 ± 11.2 years, 7 Male). Post-CDA BM trephines were collected an average of 134 days (range 52–414) after treatment. BM trephines from 10 patients undergoing staging investigations for lymphoma but without BM involvement were included as controls (mean age at time of sample 50.6 ± 13.4 years, 8 Male). At the time of sample collection BM trephines were processed using standard diagnostic laboratory practice (fixation in B5, decalcification in acid and paraffin embedding). This analysis of archival samples left over from diagnostic procedures was approved under a waiver of consent by the Melbourne Health Human Research Ethics Committee and conducted in accordance with the Declaration of Helsinki.

Clinical data was obtained from review of patient records (Supplementary Table 1). For correlation with response to treatment, patients were classified based on the need for CDA re-treatment into either durable responders (long term response without need for CDA retreatment; duration of response 9.1 ± 4.1 years, n = 4) and non-durable responders (short term response requiring CDA re-treatment, duration of response 2.25 ± 1.3 years, n = 4). One patient was excluded from response assessment as they died from causes unrelated to HCL or HCL therapy shortly after their post-treatment sample collection.

### Digital spatial profiling

From identified archival BM trephine blocks, 4 μm sections were cut and mounted on super frosted slides. DSP was performed by NanoString Technologies using the GeoMX platform as previously described (Koldej and Ritchie 2020). To focus the analysis on immune infiltrates, for each trephine 6 × 300um CD3^+^/CD45^+^ regions were selected by standard fluorescent immunohistochemistry (Fig. 1a, Supplementary Figure 2). A pre-designed GeoMX DSP panel was applied to each region to determine the expression of 57 proteins (4-1BB, ARG1, B7-H3, BCL2, Beta-2-Microglobulin, CD11c, CD127, CD14, CD163, CD20, CD25, CD27, CD3, CD34, CD4, CD40, CD44, CD45, CD45RO, CD56, CD66b, CD68, CD8, CD80, CTLA4, EpCAM, ERa, FAPa, Fibronectin, FOXP3, GAPDH, GITR, GZMB, Her2, Histone H3, HLA-DR, ICOS, IDO1, Ki-67, LAG-3, MART1, Ms IgG1, Ms IgG2a, NY-ESO-1, OX40L, PanCK, PD-1, PD-L1, PD-L2, PTEN, Rb IgG1, S100B, S6, SMA, STING, TIM-3, VISTA). Samples were analysed in 2 batches with patient HCL002 analysed in both batches to allow batch variation correction.

### Bioinformatic analyses

Data exploration and quality checks were conducted on the raw count data generated from the DSP analysis. Relative log expression (RLE) plots were used assess the presence of unwanted variation in the data^28^. Raw counts were first normalised using the ERCC positive controls and then by the trimmed mean of M-values (TMM) method^29^ using all the markers in the panel (Supplementary Figure 3). Specifically, log-transformed transcript abundance data were median-centred for each protein, and then within each sample the difference between the observed and population median of each protein was calculated. Principal components analysis (PCA) of the samples was conducted to identify variability related to specific factors in the dataset and experimental design.

Differential expression (DE) analysis was undertaken using R/Bioconductor package *limma* (v3.44.3)^30^. Based on the observed differences from the PCA analyses, considerations were made to allow for similarity that exists for regions originating from the same patient using *duplicationCorrelations* in *limma*^31^. The flexible modelling framework afforded by linear models was used to account for differences between *patient cohort*, *batch* and *patient responses* by including them as covariates in the models.

Two main covariables were investigated in this analysis: treatment *cohort* (Pre-treatment, Post-treatment and Healthy controls) and *patient response* (durable vs. non-durable). For *cohort* studies, three comparisons were modelled: *Pre-treatment versus Control*, *Post-treatment versus Control* and *Post-treatment versus Pre-treatment,* all with *batch* covariate. For *patient responses* analysis, the main comparisons undertaken was *(Pre-treatment vs. Post-treatment for Durable patients) vs. (Pre-treatment vs. Post-treatment for Non-durable)* with *batch* as a covariate. For these contrasts, the *voom-limma* with *duplicationCorrelations* pipeline^32^ was used to fit linear models. The TREAT criteria was applied^33^ (*p* value < 0.05) to perform statistical tests and subsequently calculate the t-statistics, log-fold change (logFC), and adjusted *p* values for all proteins.

## Supplementary Information

Supplementary Information.

## Data Availability

The datasets generated and/or analysed during the current study are available from the corresponding author on reasonable request subject to ethics approval.

## References

[1] Paillassa J (2020). Analysis of a cohort of 279 patients with hairy-cell leukemia (HCL): 10 years of follow-up. Blood Cancer J..

[2] Kreitman RJ (2019). Hairy cell leukemia: present and future directions. Leuk. Lymphoma.

[3] Stroup R, Sheibani K (1992). Antigenic phenotypes of hairy cell leukemia and monocytoid B-cell lymphoma: an immunohistochemical evaluation of 66 cases. Hum. Pathol..

[4] Salem DA (2019). Differential expression of CD43, CD81, and CD200 in classic versus variant hairy cell leukemia. Cytometry Part B Clin. Cytom..

[5] Tytherleigh L, Taparia M, Leahy MF (2001). Detection of hairy cell leukaemia in blood and bone marrow using multidimensional flow cytometry with CD45-PECy5 and SS gating. Clin. Lab. Haematol..

[6] Kumar P (2020). Hairy cell leukemia expresses programmed death-1. Blood Cancer J..

[7] Knight RA, Worman CP, Cawley JC (1983). Defective autologous and allogeneic mixed lymphocyte reactions in hairy cell leukaemia. Clin. Exp. Immunol..

[8] Trentin L (1990). Mechanisms accounting for the defective natural killer activity in patients with hairy cell leukemia. Blood.

[9] van de Corput L, Falkenburg JH, Kester MG, Willemze R, Kluin-Nelemans JC (1999). Impaired expression of CD28 on T cells in hairy cell leukemia. Clin. Immunol..

[10] van der Horst FA, van der Marel A, den Ottolander GJ, Kluin-Nelemans HC (1993). Decrease of memory T helper cells (CD4+ CD45R0+) in hairy cell leukemia. Leukemia.

[11] Raspadori D (1999). Long-lasting decrease of CD4+/CD45RA+ T cells in HCL patients after 2-chlorodeoxyadenosine (2-CdA) treatment. Leukemia.

[12] Matutes E (2006). Immunophenotyping and differential diagnosis of hairy cell leukemia. Hematol. Oncol. Clin. North Am..

[13] Thompson PA, Ravandi F (2017). How I manage patients with hairy cell leukaemia. Br. J. Haematol..

[14] Koldej R, Ritchie D (2020). High multiplex analysis of the immune microenvironment in bone marrow trephine samples using GeoMX^TM^ digital spatial profiling. Immuno-Oncol. Technol..

[15] Rutella S (1996). Flow cytometric detection of CD44 (H-CAM) in hairy cell leukemia. Leuk. Lymphoma.

[16] Bruck O (2020). Immune profiles in acute myeloid leukemia bone marrow associate with patient age, T-cell receptor clonality, and survival. Blood Adv..

[17] Bauer M (2021). Altered spatial composition of the immune cell repertoire in association to CD34(+) blasts in myelodysplastic syndromes and secondary acute myeloid leukemia. Cancers (Basel).

[18] Schirmer M, Hilbe W, Geisen F, Thaler J, Konwalinka G (1997). T cells and natural killer cells after treatment of hairy cell leukaemia with 2-chlorodeoxyadenosine. Acta Haematol..

[19] Juliusson G, Lenkei R, Liliemark J (1994). Flow cytometry of blood and bone marrow cells from patients with hairy cell leukemia: phenotype of hairy cells and lymphocyte subsets after treatment with 2-chlorodeoxyadenosine. Blood.

[20] Seymour JF, Kurzrock R, Freireich EJ, Estey EH (1994). 2-chlorodeoxyadenosine induces durable remissions and prolonged suppression of CD4+ lymphocyte counts in patients with hairy cell leukemia. Blood.

[21] Moser T (2020). Long-term peripheral immune cell profiling reveals further targets of oral cladribine in MS. Ann. Clin. Transl. Neurol..

[22] Stuve O (2019). Effects of cladribine tablets on lymphocyte subsets in patients with multiple sclerosis: an extended analysis of surface markers. Ther. Adv. Neurol. Disord..

[23] Mitosek-Szewczyk K (2013). Impact of cladribine therapy on changes in circulating dendritic cell subsets, T cells and B cells in patients with multiple sclerosis. J. Neurol. Sci..

[24] Kraus SH (2014). Cladribine exerts an immunomodulatory effect on human and murine dendritic cells. Int. Immunopharmacol..

[25] Van De Corput L, Falkenburg JH, Kluin-Nelemans JC (1998). T-cell dysfunction in hairy cell leukemia: an updated review. Leuk. Lymphoma.

[26] Ishikawa H, Barber GN (2008). STING is an endoplasmic reticulum adaptor that facilitates innate immune signalling. Nature.

[27] Vedantham S, Gamliel H, Golomb HM (1992). Mechanism of interferon action in hairy cell leukemia: a model of effective cancer biotherapy. Can. Res..

[28] Gandolfo LC, Speed TP (2018). RLE plots: Visualizing unwanted variation in high dimensional data. PLoS ONE.

[29] Robinson MD, Oshlack A (2010). A scaling normalization method for differential expression analysis of RNA-seq data. Genome Biol..

[30] Ritchie ME (2015). limma powers differential expression analyses for RNA-sequencing and microarray studies. Nucleic Acids Res..

[31] Smyth GK, Michaud J, Scott HS (2005). Use of within-array replicate spots for assessing differential expression in microarray experiments. Bioinformatics.

[32] Law CW, Chen Y, Shi W, Smyth GK (2014). voom: precision weights unlock linear model analysis tools for RNA-seq read counts. Genome Biol..

[33] McCarthy DJ, Smyth GK (2009). Testing significance relative to a fold-change threshold is a TREAT. Bioinformatics.

