# Supplementary material for: Dissection of the bone marrow microenvironment in hairy cell leukaemia identifies prognostic tumour and immune related biomarkers
Source: Sci Rep. 2021 Sep 24;11:19056. doi: 10.1038/s41598-021-98536-1 (PMC8463612; doi:10.1038/s41598-021-98536-1)
Supplement: Supplementary file 1 — Supplementary Information. [file 41598_2021_98536_MOESM1_ESM.pdf]

# Dissection of the bone marrow microenvironment in Hairy Cell Leukaemia identifies prognostic tumour and immune related biomarkers

Rachel M Koldej[1,2*], Ashvind Prabahran[1,2,3], Chin Wee Tan[4,5], Ashley P. Ng[3,4], Melissa J Davis[4,5,6] and David S Ritchie[1,2,3]

[1]ACRF Translational Research Laboratory, Royal Melbourne Hospital, Melbourne, Australia, [2]Department of Medicine, Faculty of Medicine, Dentistry and Health Sciences, University of Melbourne, Melbourne, Australia, [3]Clinical Haematology, Peter MacCallum Cancer Centre and Royal Melbourne Hospital, Melbourne, Australia, [4]The Walter and Eliza Hall Institute of Medical Research, Parkville, Melbourne, Australia, [5]Department of Medical Biology, Faculty of Medicine, Dentistry and Health Sciences, University of Melbourne, Melbourne, Australia,[6]Department of Clinical Pathology, Faculty of Medicine, Dentistry and Health Sciences, University of Melbourne, Melbourne, Australia.

## Supplementary data

## Supplementary Table 1 – Patient Characteristics

| Pt ID | Sex | Age at diagnosis | Clinical Response | | % cellularity* | | BM aspirate % lymphocytes | | PB lymphocyte count | | PB %CD3 | | PB %CD19 | |
|---|---|---|---|---|---|---|---|---|---|---|---|---|---|---|
| | | | Duration (yrs) | Classification | Pre CDA | Post CDA | Pre CDA | Post CDA | Pre CDA | Post CDA | Pre CDA | Post CDA | Pre CDA | Post CDA |
| HCL001 | M | 39 | 4 | Non-Durable | Hyper 90-100% | Hyper 40-90% | NP | 8 | 2.1 | 0.7 | 75 | 15 | 11 | 9 |
| HCL002 | M | 48 | 2 | Non-Durable | Hyper 50-70% | Hyper 60-80% | 12.5 | 7.5 | 1.1 | 0.5 | 58 | 24 | 33 | 6 |
| HCL003 | M | 66 | 7 | Durable | Hyper 80-100 | Norm 40-60% | NP | 6.5 | 1.1 | 2.2 | 16 | 92 | 84 | 2 |
| HCL004 | F | 53 | 5 | Durable | Hyper 70% | Norm 50% | 16.5 | 7 | 0.9 | 0.5 | 27 | 80 | 70 | 9 |
| HCL005 | M | 33 | 2 | Non-Durable | Hyper 60-80% | Norm 50-70% | NP | 13 | 13.1 | 0.7 | 0 | 30 | 99 | 25 |
| HCL006 | M | 43 | 13 | Durable | Hyper 90-100% | Hypo 25-35% | NP | 29.5 | 2.2 | 1 | 59 | 90 | 34 | 1 |
| HCL007 | M | 57 | 13 | Durable | Norm 40-60% | Norm 40-60% | 18 | 4 | 0.8 | 0.4 | 92 | 85 | 6 | 4 |
| HCL008 | F | 49 | NA | - | Hypo 5-10% | Hypo 10-50% | 39 | 12 | 0.7 | 0 | 87 | 95 | 12 | 1 |
| HCL009 | M | 65 | 1 | Non-Durable | Hyper 60-80% | Norm 30-70% | 32.5 | 30.5 | 1 | 1.2 | 73 | 73 | 5 | 5 |
| HC1 | M | 64 | - | - | Norm 50% | - | 6 | - | 1.5 | - | NP | - | NP | - |
| HC2 | M | 44 | - | - | Norm 50% | - | 10 | - | 1.7 | - | NP | - | NP | - |
| HC3 | F | 56 | - | - | Norm 50% | - | 9.5 | - | 0.7 | - | NP | - | NP | - |
| HC4 | M | 33 | - | - | Norm 50% | - | 10 | - | 2.1 | - | NP | - | NP | - |
| HC5 | M | 32 | - | - | Norm 60-70% | - | 12 | - | 0.6 | - | NP | - | NP | - |
| HC6 | M | 58 | - | - | Hyper | - | 6 | - | 2.2 | - | 67 | - | 5 | - |
| HC7 | F | 63 | - | - | Hypo 40% | - | 22 | - | 2.8 | - | 81 | - | 14 | - |
| HC8 | M | 37 | - | - | Norm | - | 21.5 | - | 1.5 | - | 71 | - | 22 | - |
| HC9 | M | 51 | - | - | Norm 50% | - | 6.5 | - | 1.5 | - | 74 | - | 20 | - |
| HC10 | M | 68 | - | - | Norm | - | 6.5 | - | 2.3 | - | NP | - | NP | - |

NA - patient died of unrelated causes prior to response

NP - test not performed

*determined by independent pathologist review

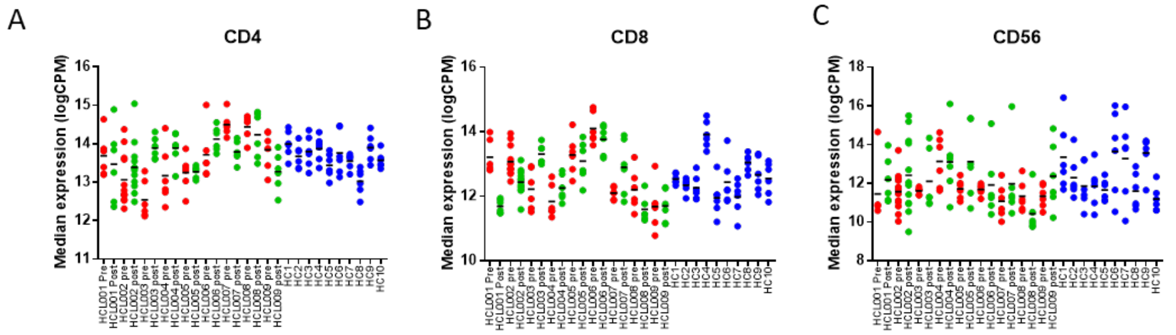


**Supplementary Figure 1** – CDA does not affect the relative expression of CD4 (A), CD8 (B) or CD56 (C)

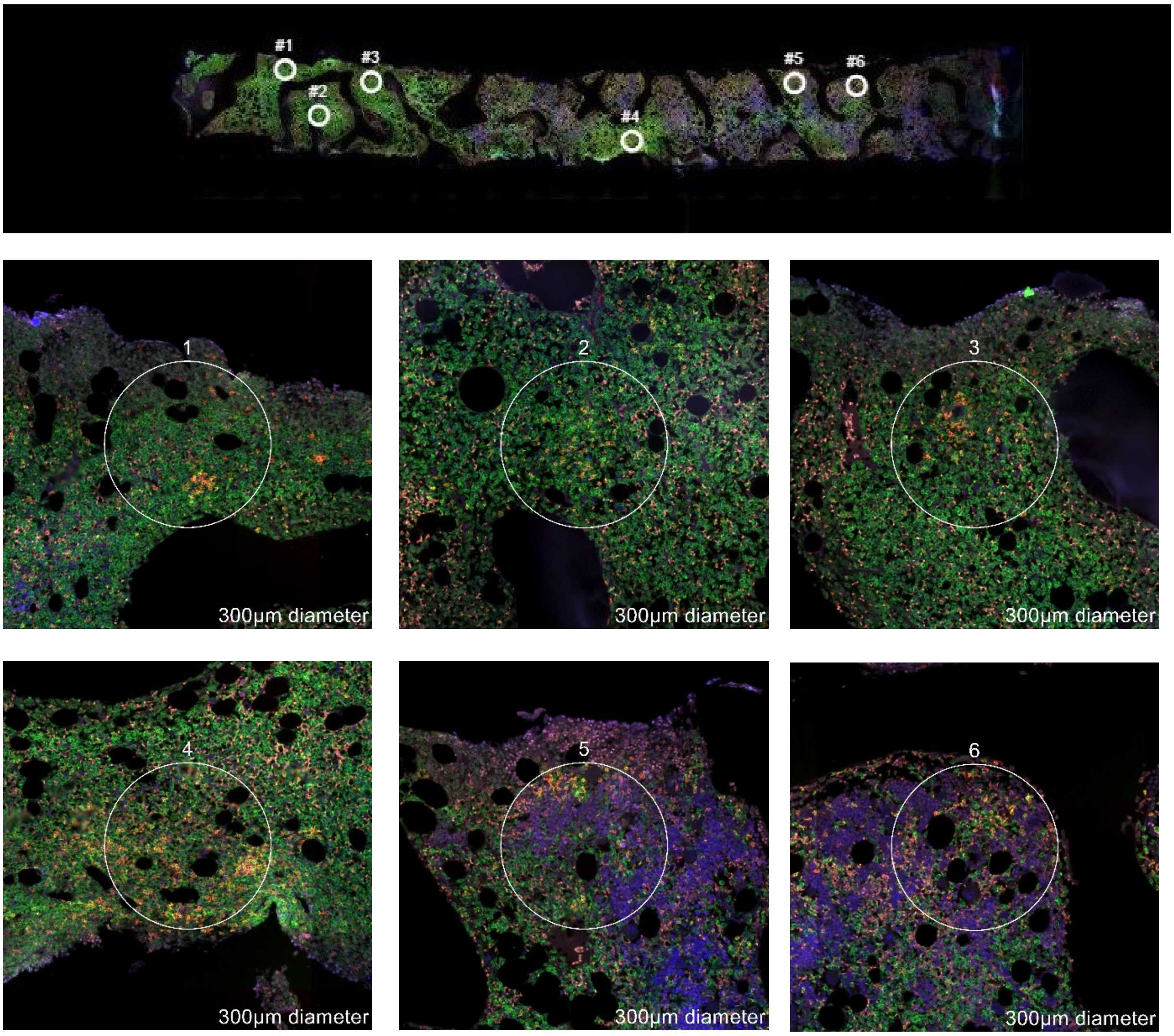


**Supplementary Figure 2** - Representative HCL pre-CDA BM trephine showing distribution of ROIs within the tissue focused on areas of high CD3/45 staining in a background of high CD45 tumour burden. Red = CD3, Green = CD45, Blue = Nuceli, Yellow = dual CD3/CD45 positive cells

A

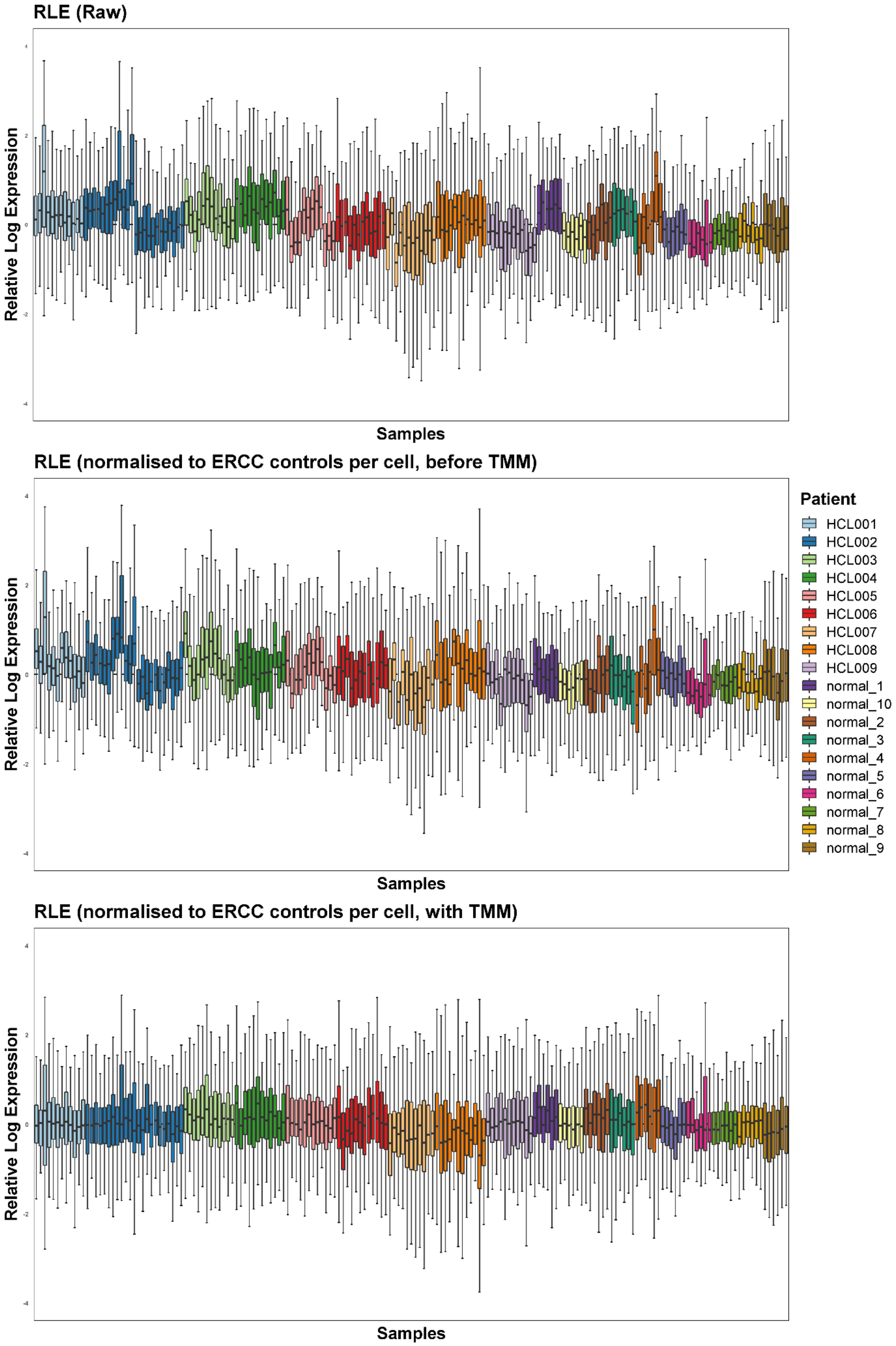
RLE (Raw)
Relative Log Expression
Samples
RLE (normalised to ERCC controls per cell, before TMM)
Relative Log Expression
Samples
RLE (normalised to ERCC controls per cell, with TMM)
Relative Log Expression
Samples
Patient
HCL001
HCL002
HCL003
HCL004
HCL005
HCL006
HCL007
HCL008
HCL009
normal_1
normal_10
normal_2
normal_3
normal_4
normal_5
normal_6
normal_7
normal_8
normal_9

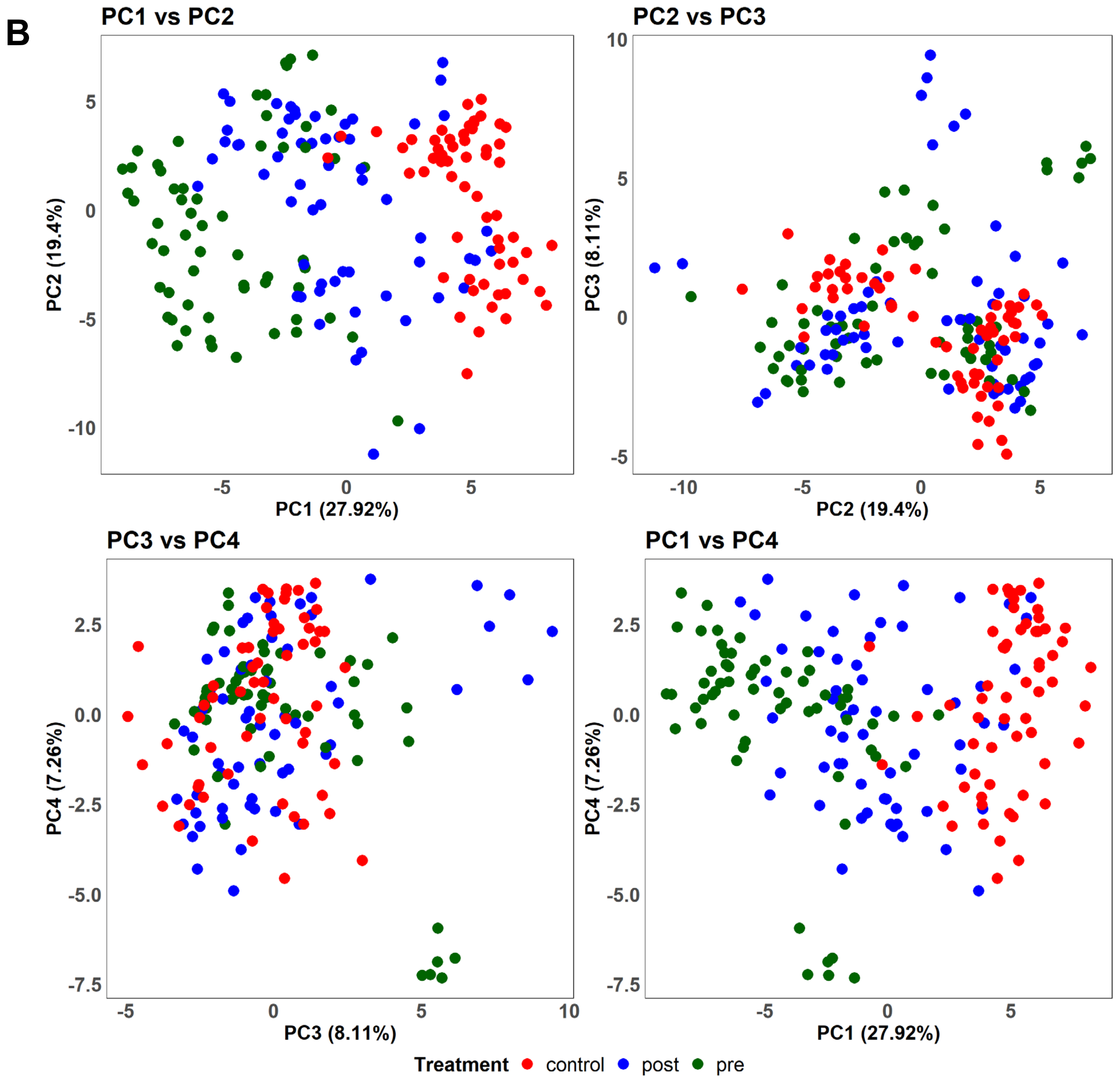


**Supplementary Figure 3** – Data normalisation and diagnostics

**(A)** Relative log expression (RLE) plots for (**top**) raw samples, (**middle**) normalised to ERCC controls per cell and (**bottom**) normalised to ERCC controls per cells and using the trimmed mean of M-values (TMM) method using the all the markers in the panel. The sequential normalisation procedures removed the systematic bias in the dataset. TMM normalisation was conducted using the calcNormFactors function in R package edgeR. **(C)** Principal components (PCs) plots capturing orthogonal dimensions of variability in the data in descending order of contribution (i.e. PC1-PC2, PC3-

PC2, PC1-PC4 and PC3-PC4) were plotted stratifying based on the treatment factor in the experimental data. The treatment effect observed along the dominant PC1.

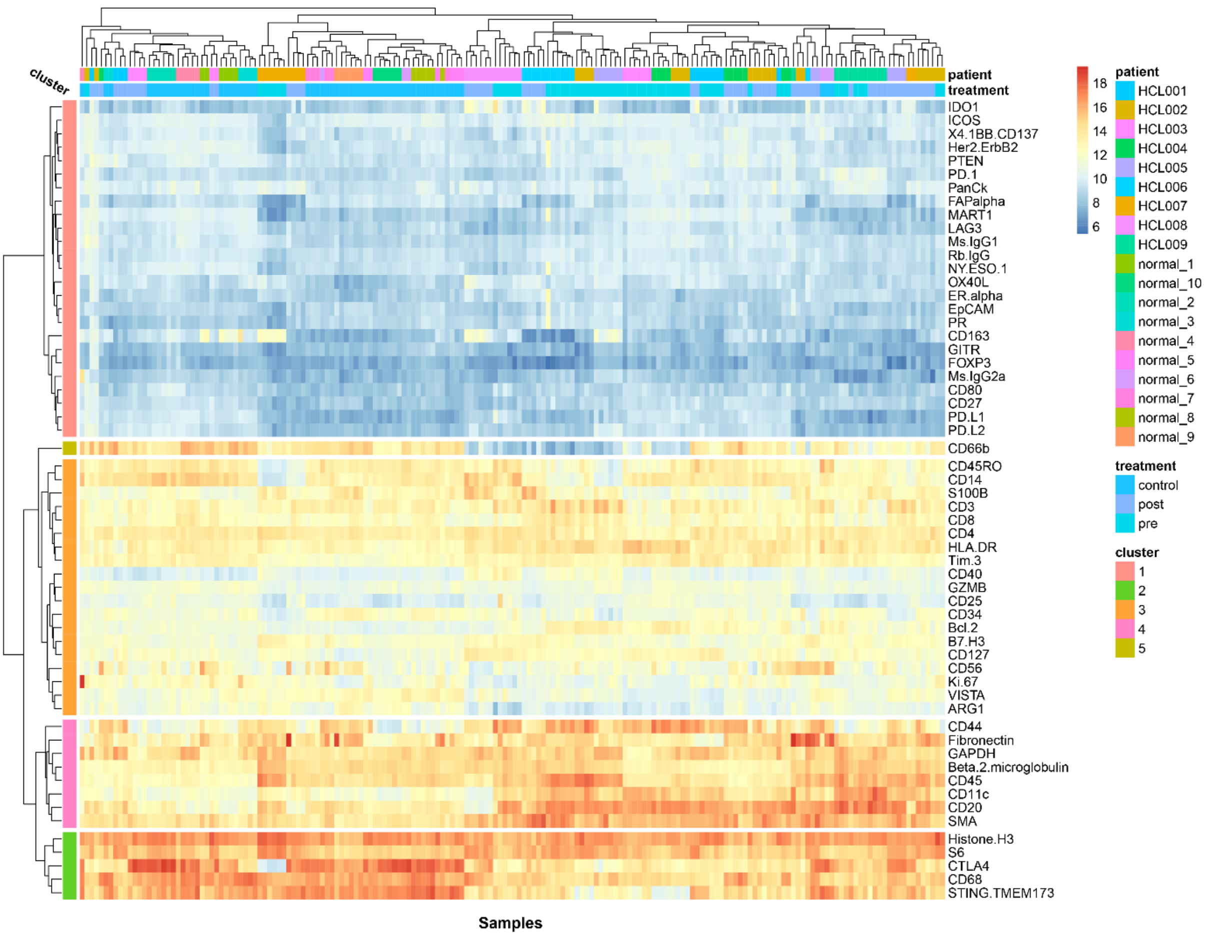


**Supplementary Figure 4** – Heatmap of entire dataset

Heatmap generated using the logCPM values of the entire dataset, visualising the z-score values across each sample. Samples on the right axis and marker panel on the y-axis. Hierarchical clustering applied on both the samples and marker as shown by the respective dendrograms. Heatmaps were generated in R (version 1.0.12) using R package pheatmap. Hierarchical clustering was conducted using the hclust function in the base R statistics package
